# The effects of 1,4-dimethylpyridine in metastatic prostate cancer in mice

**DOI:** 10.1186/s12885-017-3161-4

**Published:** 2017-03-07

**Authors:** Agnieszka Denslow, Marta Switalska, Marcin Nowak, Magdalena Maciejewska, Stefan Chlopicki, Andrzej Marcinek, Jerzy Gebicki, Joanna Wietrzyk

**Affiliations:** 1 Hirszfeld Institute of Immunology and Experimental Therapy, Polish Academy of Sciences, Weigla 12, Wroclaw, 53-114 Poland; 2 Wroclaw University of Environmental and Life Sciences, Norwida 31, Wroclaw, 50-375 Poland; 3 Chair of Pharmacology, Jagiellonian University, Medical College, Grzegorzecka 16, Krakow, 31-531 Poland; 4 Jagiellonian Center for Experimental Therapeutics (JCET), Jagiellonian University, Bobrzynskiego 14, Krakow, 30-348 Poland; 5 Lodz University of Technology, Zeromskiego 116, Lodz, 90-924 Poland

**Keywords:** Prostate cancer, Metastasis, Prevention, Combined therapy, 1-methylnicotinamide, 1,4-dimethylpyridine, Docetaxel

## Abstract

**Background:**

We previously showed that 1-methylnicotinamide (1-MNA) and its analog 1,4-dimethylpyridine (1,4-DMP) could inhibit the formation of lung metastases and enhance the efficacy of cyclophosphamide-based chemotherapy in the model of spontaneously metastasizing 4T1 mouse mammary gland tumors. In the present study, we aimed to investigate whether the previously observed activity of pyridine compounds pertains also to the prevention and the treatment of metastatic prostate tumors, in a combined chemotherapy with docetaxel.

**Methods:**

Cancer-preventing activity of 1,4-DMP was studied in the model of prostate tumors spontaneously arising in C57BL/6-Tg (TRAMP)8247Ng/J (TRAMP) mice. The efficacy of the combined chemotherapy, comprising simultaneous use of 1,4-DMP and docetaxel, was evaluated in the orthotopic mouse model of human PC-3M-luc2 prostate cancer. The toxicity of the applied treatment was also determined.

**Results:**

The development of prostate tumors in TRAMP mice remained unaffected after administration of 1,4-DMP. Similarly, no effect of 1,4-DMP was found on the growth of orthotopically transplanted PC-3M-luc2 tumors. However, when 1,4-DMP was administered along with docetaxel, it enhanced the anticancer activity of the chemotherapy. As a result, in PC-3M-luc2-bearing mice statistically significant inhibition of the tumor growth and lower metastases incidence were observed. The decreased metastatic yield is probably related to the diminished platelet activity observed in mice treated with combined therapeutic regimen. Finally, the combined treatment exhibited lowered side effects accompanying docetaxel administration.

**Conclusions:**

Results presented herein confirm previously published data on the anticancer activity of pyridine compounds and demonstrate that 1,4-DMP may be beneficially implemented into chemotherapy utilizing various cytotoxic agents, directed against multiple metastatic tumor types.

**Electronic supplementary material:**

The online version of this article (doi:10.1186/s12885-017-3161-4) contains supplementary material, which is available to authorized users.

## Background

Prostate cancer is the second most common cancer of men, affecting approximately 14% of patients [1]. While the risk of developing prostate cancer might be beneficially influenced by proper diet and physical activity [2], there are no confirmed pharmacological means for the prevention of these types of tumors. The majority of prostate cancer cases are diagnosed at the localized stage enabling effective treatment; however, a significant fraction of patients develops metastatic disease that often progresses into treatment-irresponsive, ultimately resulting in patient’s death [3].

Initial treatment of prostate cancer usually comprises hormone therapy; however, when tumors are irresponsive to hormonal treatment (i.e., in case of castrate-resistant prostate cancer), the most common first-line treatment includes the simultaneous use of docetaxel and prednisone. Docetaxel is a semi-synthetic taxane that inhibits microtubular depolymerization and block *bcl-2* and *bcl-xl* gene expression [4, 5]. Prednisone, in turn, is a glucocorticoid that is used to improve symptoms such as pain [6]. It was also shown to inhibit cell proliferation and induce apoptosis in prostate cancer cells [7, 8], and thus decrease the level of prostate-specific antigen [9]. Accordingly, in multiple studies, prednisone was shown to promote anticancer activity of docetaxel [10–14]. However, the use of glucocorticosteroids in patients with prostate cancer is associated with the risk of adverse side effects (as reviewed, for example, by Dorff and Crowford [15]), and it eventually leads to the development of resistance to chemotherapy [16]. Therefore, there is still an urgent need for new treatment regimens that would enable efficient yet safe means for the therapy of patients suffering from prostate cancer.

1-methylnicotinamide (1-MNA) is an endogenous metabolite of nicotinamide (NA) that has recently gained attention due to its anti-inflammatory [17] and anti-thrombotic [18] activity driven by mechanisms dependent on prostacyclin (PGI_2_) release [18, 19]. Another compound that has been shown to modulate thrombus formation based on the PGI_2_-related mechanisms is 1,4-dimethylpyridine (1,4-DMP) – a structural analog of 1-MNA that arises naturally in roasted coffee seeds [20]. In addition, it has been recently shown that both 1-MNA and 1,4-DMP could inhibit metastases formation in the model of experimental and spontaneous metastasis of 4T1 murine mammary gland cancer [21].

The present work is aimed to establish whether 1,4-DMP may have an anti-oncogenic effect in the prophylaxis and the treatment of prostate tumors.

## Methods

### Drugs

1,4-DMP and 1-MNA were used in the form of chlorides provided by the Institute of Applied Radiation Chemistry, Technical University of Lodz, Poland. Prior to use, both salts were diluted in drinking water such that mice received the predetermined dose of the drugs. Docetaxel (DTX) was purchased at Ak Scientific (USA). All drugs were administrated at the doses and according to the schedules presented in Table 1.

**Table 1:** Drugs, doses and therapeutic regimens applied in the presented studies

Experimental model	Drug	Route of administration	Dose	Treatment regimen
Spontaneous tumor formation (Fig. 1)	1-MNA	per os in drinking water	100 mg/kg/day	continuously from the age of 8 to 12 weeks to the day of the necropsy
	1,4-DMP	per os in drinking water	100 mg/kg/day	continuously from the age of 8 to 12 weeks to the day of the necropsy
PC-3 M-luc2 tumors (Fig. 2)	1,4-DMP	per os in drinking water	100 mg/kg/day	continuously from the day 1 to the end of the experiment
	DTX	intraperitoneally	10 mg/kg	2 doses at days 15 and 22

### Mice

Eight- to twelve-weeks-old male C57BL/6-Tg(TRAMP) 8247 Ng/J (TRAMP) mice were purchased from the Jackson Laboratory (USA). Seven- to eight-weeks-old BALB/c Nude male mice were provided by Charles Rivers Laboratories (Germany) (Table 2). All experiments were performed according to the *Interdisciplinary Principles and Guidelines for the Use of Animals in Research, Marketing and Education* issued by the New York Academy of Sciences’ Ad Hoc Committee on Animal Research and were approved by the 1st Local Committee for Experiments with the Use of Laboratory Animals, Wroclaw, Poland.

**Table 2:** Strains and number of mice used in the experiments

Experimental model	Mouse strain	No of mice/group	Total No of used mice
Spontaneous tumor formation (Fig. 1)	TRAMP	15	45
PC-3M-luc2 tumors (Figs. 2, 3 and 4)	C57BL/6	9	36

### Cell culture and transplantation

Human prostate cancer PC-3M-luc2 cell line stably expressing the firefly luciferase gene (*luc*) was obtained from Caliper Life Sciences Inc. (USA). Cells were cultured in RPMI 1640 + Gluta-MAX™ medium (Life Technologies, USA) supplemented with 10% fetal bovine serum (Sigma-Aldrich, Germany) and antibiotics (penicillin and streptomycin—Polfa Tarchomin, Poland). Cell line cultures were maintained at 37 °C in a humidified atmosphere with 5% CO_2_.

Prior to the transplantation, cells were trypsinized (IIET, Poland), centrifuged (200 × g, 4 °C, 5 min) and counted. Then, cells were resuspended in Hank’s Balanced Salt Solution (HBSS; IIET, Poland).

Male BALB/c Nude mice were intraperitoneally injected with ketamine at a dose of 50 mg/kg (VET-AGRO Sp. z o.o., Poland) and anesthetized with the mixture of air and isoflurane (3% v/v). 1.0 cm wide abdominal wall incision was made just above the bladder, in the lower part of abdomen, and the prostate gland was exposed for the injection. Then, 5 × 10^6^ PC-3M-luc2 cells in 0.05 ml of HBSS were inoculated into the dorsal prostate lobes of mice. Immediately after the transplantation, incised abdominal wall and skin were sewed with soluble surgical suture.

### Estimation of the antitumor activity

The development of prostate tumors in TRAMP mice was monitored weekly by physical examination. Adenocarcinoma formation was confirmed by histological examination of the tumors isolated from mice during necropsy carried out in animals with clear physiological (e.g., body weight or body temperature decrease, body posture, ruffled fur) and behavioral symptoms (e.g., decreased movement) of an advanced disease. Briefly, prostate tumors were isolated and fixed in buffered formalin, and then cut into 4-μm-thick sections that were subsequently dewaxed with xylene. Following rehydration in a gradient of ethanol, the sections were washed in distilled water, cytoplasm was stained with eosin while nuclei were counterstained in hematoxylin. Finally, the preparations were dehydrated in an alcohol gradient and coverslip mounted. The histological appearance of the tissue was examined at 50× or 100× magnitude.

Using an In vivo MS FX PRO system (Carestream Health INC., USA), in vivo visualizations of PC-3 M-luc2 tumors growing in prostate gland of BALB/c Nude mice were performed no more often than every 4 days starting from the 15th day of the experiment. In brief, about 10 min before imaging, D-luciferin potassium salt (Synchem INC., Germany) was administered to each mouse intraperitoneally at a dose of 150 mg/kg. Then, animals were anesthetized with a 3–5% (v/v) mixture of isoflurane (Forane, Abbott Laboratories, USA) in synthetic air (200 ml/min). Anesthesia was maintained with 1.5–2% (v/v) mixture of isoflurane and synthetic air delivered via individual masks. Visualization was carried out using the following settings: for X-ray — t = 2 min, f-stop = 5.57, FOV = 198.6; for luminescence capture — t = 3 min, binning 2 × 2, f-stop = 5.57, FOV = 198.6. Images were analyzed with Carestream MI SE software (Carestream Health INC., USA). The intensity of the luminescent signal is presented as the sum intensity of the region of interest and expressed in arbitrary units (a.u.). Tumor tissue was also excised and weighted on the last day of the experiment (day 46).

### Evaluation of the antimetastatic effect

Livers, lungs, kidneys, bones and axillary as well as inguinal lymph nodes were isolated and fixed in buffered formalin on the day of the necropsy, in order to detect metastases in the mice bearing prostate tumors. Then, tissue samples were cut into 4-μm-thick sections and stained as described hereinabove. The number of metastases in isolated tissues was counted at 50× or 400× magnitude.

### Platelet activation status

Blood samples were collected on days 87, 122, 213 and during animal’s necropsy in the model of the spontaneously formed prostate tumors or on the last day of the experiment (day 46) in case of mice bearing PC-3M-luc2 tumors. Samples were collected in tubes containing 0.05 ml of 5% ethylenediaminetetraacetic acid (EDTA) solution (Sigma-Aldrich, Germany). Platelet-related morphology analyzes were performed using Mythic 18 analyzer (C2 Diagnostics, France). Then, blood plasma was obtained by centrifugation (2000 × g, 15 min, 4 °C) and stored at −80 °C until further analyzes. Prostacyclin generation in the treated mice was determined by the quantification of plasma 6-keto-prostaglandin F1α (6-keto-PGF1α) levels. Based on thromboxane B_2_ (TXB_2_), von Willebrand factor (vWF) and soluble P-selectin plasma concentrations, platelet activation status was estimated. Using commercial kits available from Cusabio Biotech Co. Ltd. (Wuhan, China), all analyzes were conducted via the ELISA technique. In addition, plasma concentration of transforming growth factor β1 (TGF-β1) was determined with ELISA kit from Boster Biological Technology (USA). All ELISA-based analyzes were conducted according to the manufacturer’s instructions.

### Protein expression in tumor tissue

Protein expression in prostate tumor tissue was analyzed according to the standard Western blot procedure [22]. In brief, using a FastPrep®-24 MP Bio device (Mp Biomedicals LLC., USA), samples of tumor tissue that were collected and immediately frozen on the last day of the experiments were homogenized in RIPA Buffer (Sigma-Aldrich, Germany) with the following settings: CP 24 × 2, 6 m/s, 40 s. According to the manufacturer’s protocol, protein content in all samples was analyzed using a Bio-Rad Protein Assay (Bio-Rad Laboratories Inc., USA). Samples containing 100 μg of protein were separated on the pre-cast 4–20% gradient gels (Bio-Rad Laboratories, Inc., USA) and transferred onto 0.45 μm polyvinylidene fluoride (PVDF) membranes (Merck Millipore, USA). Next, the membranes were probed with primary rabbit polyclonal anti-E-cadherin (1:1000), anti-N-cadherin (1:1000), anti-VEGFR-1 (1:200) antibodies (all from Proteintech Group, USA) or mouse anti-β-actin (1:1000, Sigma-Aldrich, Germany) antibody. Finally, according to the manufacturer’s instruction, the analyzed proteins were detected with IRDye® 800CW Goat anti-Rabbit IgG or IRDye® 680RD Donkey anti-Mouse IgG (both from LI-COR, USA). Blots were visualized in ODDYSEY® CLx Imager (LI-COR, USA) and analyzed with ImageJ Software as follows. The total E-cadherin cellular content comprising truncated and unprocessed E-cadherin (with a molecular weight of approximately 100 and 130 kDa, respectively) was calculated. Similarly, total N-cadherin cellular content comprising mature and unprocessed N-cadherin (with a molecular weight of approximately 70 and 100 kDa, respectively) was determined. Then, E-cadherin and N-cadherin contents were normalized to β-actin. Finally, E-cadherin to N-cadherin ratios in individual samples were calculated and presented as mean ± SD values.

### Toxicity of the anticancer treatment

The toxicity of the proposed anticancer treatment strategy and its influence on the overall health condition were estimated based on body weight changes as well as morphological and biochemical blood analyzes. The body weight of experimental animals was measured thrice each week throughout the course of all studies.

Blood morphology was performed with Mythic 18 analyzer (C2 Diagnostics, France). Using reagents and procedures provided by the manufacturer, biochemical analyzes were performed in Cobas C 111 analyzer (Roche Diagnostics, Switzerland).

### Statistical analysis

Data normality was estimated using the Shapiro-Wilk test with a predetermined value of *p* < 0.05. The Tukey-Kramer multiple comparison test for parametric data or the Kruskal–Wallis Test for non-parametric data was applied; *p* values lower than 0.05 were considered significant. All calculations were performed using GraphPad Prism 7 (GraphPad Software, Inc., USA) software.

Unless stated otherwise, all data presented on graphs correspond to mean ± SD values.

## Results

### The influence of 1,4-DMP on the onset and metastasis of spontaneously formed prostate tumors

**Fig. 1:**
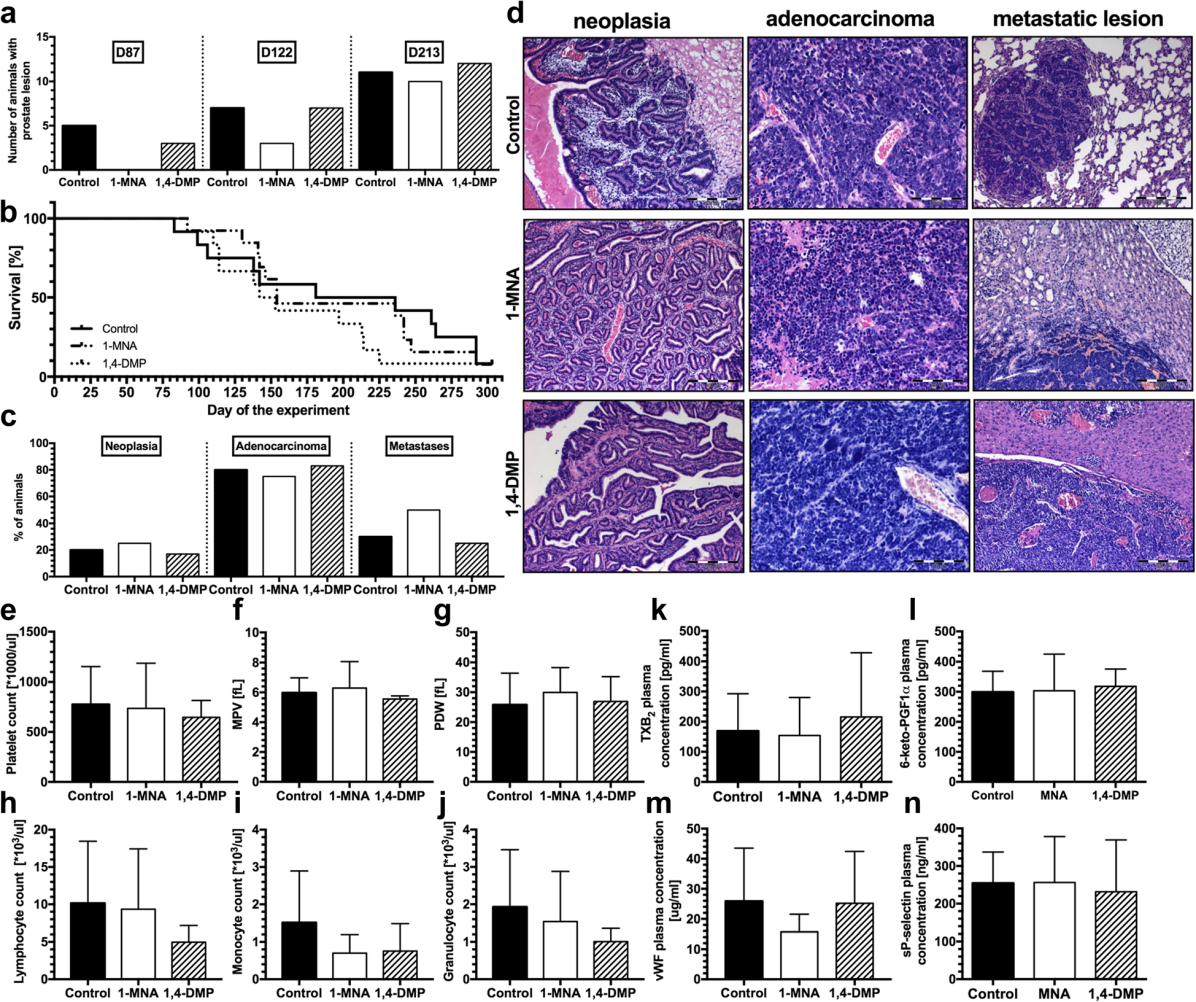
The influence of 1,4-DMP and 1-MNA on the development and the progression of prostate cancer. **a** The onset of the prostate gland lesions; **b** survival of the TRAMP mice continuously treated with 1-MNA and 1,4-DMP. **c** Summarized results of the frequency of the neoplasia, adenocarcinomas and metastases in TRAMP mice determined by the means of histopathological analysis of the tissues isolated during the necropsy. **d** Images of primary tumors (neoplasia and adenocarcinomas) identified in non-treated and drug-receiving animals and of the metastases localized in lungs of the control animals, kidney of the animal treated with 1-MNA, or liver of the mouse receiving 1,4-DMP. Results of the morphological analysis carried out on blood samples collected during the necropsy of the animals: **e** platelet count; **f** mean platelet volume (MPV); **g** platelet distribution width (PDW); **h** lymphocyte count; **i** monocyte count; and **j** granulocyte count. Plasma concentrations of **k** TXB_2_, **l** 6-keto-PGF1α, **m** vWF, **n** soluble P-selectin determined by ELISA. All data are presented as mean ± SD

To establish whether 1,4-DMP might prevent the development of prostate tumors, the compound was continuously given to male TRAMP mice that during their life span spontaneously develop mild intraepithelial hyperplasia to malignant neoplasia within prostate gland. For comparative purposes, another group of the animals was treated with 1-MNA, a primary analog of 1,4-DMP that was proven to possess significant anti-thrombotic and anti-inflammatory activity. 1-MNA, and to a lesser extent also 1,4-DMP, delayed the onset of prostate lesions in TRAMP mice (Fig. 1a). However, none of the given compounds prolonged the life span of treated animals (Fig. 1b). Histopathological analysis of the tumor tissues excised during the necropsy confirmed the development of malignant adenocarcinomas in approximately 80% of the mice in all experimental groups (Fig. 1c and d).

Histopathological analysis demonstrated that metastases in TRAMP mice prostate adenocarcinomas were developed in lymph nodes, lungs, liver and kidneys (Fig. 1d). Metastatic lesions were diagnosed in around 30% of untreated animals. Similarly, metastases were found in 30% of animals treated with 1,4-DMP. In contrast, when treated with 1-MNA, 50% of mice developed metastases (Fig. 1c, not statistically significant difference).

The analysis of morphological features of blood platelets did not reveal changes in platelet mean volume and platelet distribution width (PDW) occurring during the study; however, we noted an increase in platelet count and decreased level of PDW in animals during the necropsy (Additional File: Figure S1c). The treatment either with 1-MNA or 1,4-DMP did not affect the platelet morphological parameters (Fig. 1e–g, Additional File 1: Figure S1a-c). Similarly to platelets, red blood cell parameters also remained unaffected by the tumor progression, with an exception of the time preceding necropsy where a significant drop in red blood count was observed in all experimental groups. However, none of the studied compounds influenced the red blood cell parameters (Additional File 1: Figure S1d).

While there was no obvious change in the white blood cell count in TRAMP mice developing prostate tumors that was observed in the course of the study, we observed that 1,4-DMP and to a lesser extent 1-MNA tended to decrease the number of all of lymphocytes, monocytes and granulocytes in the treated animals, when compared to the control group (Fig. 1h–j, Additional File 1: Figure S1e–g). This effect might be attributed to prostacyclin-dependent splenic dilation in the treated mice that leads to white blood cells pooling in the spleen, and in consequence, a systemic decrease in the white blood cell count [23]. The analysis of the biochemical parameters of platelet activity revealed no effect of the studied compounds on platelet activity in TRAMP mice (Fig. 1l–o). Finally, during the tumor development process, we observed that in TRAMP mice, the plasma level of TGF-β1 was not affected either by 1,4-DMP or by 1-MNA (Additional File 1: Figure S1h).

### Anticancer activity of the combined treatment of prostate cancer comprising simultaneous application of 1,4-DMP and docetaxel

**Fig. 2:**
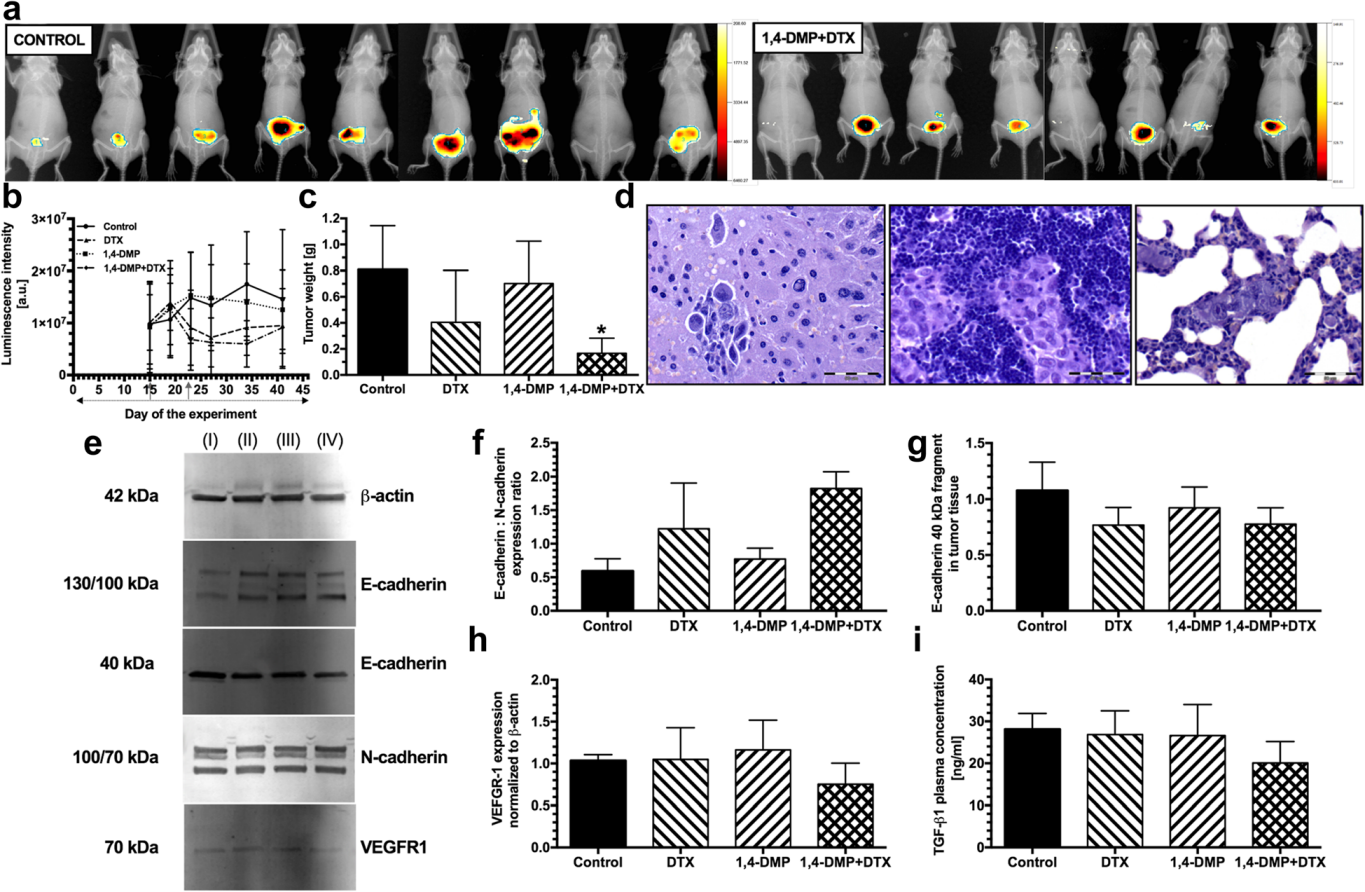
Anticancer activity of the combined treatment comprising the use of docetaxel (DTX) and 1,4-DMP in the model of human prostate cancer PC-3M-luc2 xenografted into prostate glands of BALB/c Nude mice. **a** Results of in vivo imaging of PC-3M-luc2 tumors performed on day 41 of the experiment. **b** Kinetics of the PC-3M-luc2 tumor growth in mice treated with docetaxel (DTX) and 1,4-DMP given either alone and in a comparison to the control group of animals. Days of drug administration are indicated with *gray arrows* for docetaxel (DTX) and *dotted arrow* for 1,4-DMP. **c** PC-3M-luc2 tumor weight measured on the last day of the experiment (day 46) (*p < 0.05 vs. control and 1,4-DMP). **d** Images of metastases localized in liver of the control animal, lymph node of docetaxel (DTX)-treated mouse and lungs of the 1,4-DMP-treated mouse (from left to right). **e** Images of bands obtained during Western blot analysis of protein expression in tumor tissue of (I) control animals and animals treated with (II) docetaxel (DTX), (III) 1,4-DMP and (IV) docetaxel (DTX) with 1,4-DMP. **f** E-cadherin : N-cadherin expression ratios in the samples of tumor tissue collected on the last day of the experiment. The total cellular content of E-cadherin (comprising protein characterized by the molecular weight of 130 and 100 kDa) and N-cadherin (comprising protein characterized by the molecular weight of 100 and 70 kDa) was first normalized to the content of β-actin and then used to determine E-cadherin to N-cadherin expression ratios. **g** The level of low molecular weight fragment of E-cadherin in PC-3M-luc2 tumors normalized to the content of β-actin. **h** The expression of VEGFR-1 in PC-3M-luc2 tumors normalized to the content of β-actin. **i** Plasma concentration of TGF-β1 in mice bearing PC-3M-luc2 tumors. All data are presented as mean ± SD values

The growth of primary tumors localized in prostate glands of BALB/c Nude mice was monitored throughout the experiment by in vivo imaging of the luminescence generated by PC-3M-luc2 cells. The analysis of the luminescence intensity indicated that 1,4-DMP when administered alone did not inhibit the growth of PC-3M-luc2 prostate tumors. On the contrary, marked tumor growth inhibition was observed when mice were treated with docetaxel alone or administered with 1,4-DMP (Fig. 2a and b). These observations were confirmed by the analysis of the tumor mass isolated from the mice on day 46 of the experiment. Docetaxel, when administered alone, inhibited the growth of PC-3M-luc2 tumors in around 50% when compared to the control group of animals (0.40 g vs. 0.81 g, respectively). Antitumor activity of docetaxel was additionally enhanced when the drug was administrated with 1,4-DMP and reached approx. 80% tumor growth inhibition (0.17 g vs. 0.81 g, *p* < 0.05) (Fig. 2c).

Histopathological analysis of tissues collected from mice led to the identification of PC-3M-luc2 metastatic lesions in such tissues as lymph nodes, liver and lungs (Fig. 2d). We observed that the frequency of metastases formation in mice treated with 1,4-DMP decreased by almost 50%. Similarly, the number of metastases-bearing animals decreased in case of single-drug treatment with docetaxel. Most interestingly, none of the mice treated with 1,4-DMP and docetaxel developed PC-3M-luc2 metastases during the study (see Table 3).

**Table 3:** Frequency of metastases formation and localization of new lesions in mice bearing PC-3M-luc2 tumors

Treatment	Animals with metastases/all animals tested	Metastases location
Control	7/9	Lungs, liver, lymph nodes
1,4-DMP	4/9	Lungs, liver
DTX	1/6	Lymph nodes
1,4-DMP + DTX	0/8	—

We also decided to investigate the influence of applied treatment on the metastatic potential of tumor-forming cancer cells. To this end, we evaluated the expression of E-cadherin, N-cadherin and vascular endothelial growth factor receptor 1 (VEGFR1) in tumor tissue. The results of the Western blot analysis show that 1,4-DMP had no significant effect on E-cadherin to N-cadherin expression ratio. In contrast, when mice were treated with docetaxel given alone, over twofold increase in E-cadherin to N-cadherin expression ratio was observed (not statistically significant). Such a phenomenon was additionally enhanced by simultaneous application of 1,4-DMP that allowed to reach over threefold enhancement of E-cadherin to N-cadherin expression ratio (Fig. 2f). In addition, in PC-3M-luc2 tumor-bearing mice, we have observed the appearance of low molecular weight fragments of E-cadherin (40 kDa). The concentration of these protein fragments was lowered in both groups treated with docetaxel, given either alone or with 1,4-DMP, while it was unaltered in mice receiving 1,4-DMP alone (Fig. 2g). The expression of VEGFR-1 was decreased in about 30% in mice treated with combined therapy but was not affected either by docetaxel or by 1,4-DMP given alone (Fig. 2h).

Increased E-cadherin to N-cadherin expression ratio was accompanied by the decreased plasma concentration of TGFβ-1 in mice receiving the combined treatment consisting of docetaxel and 1,4-DMP (20.5 ± 5.1 ng/ml vs. 28.13 ± 3.7 ng/ml in the control group of mice, Fig. 2i).

**Fig. 3:**
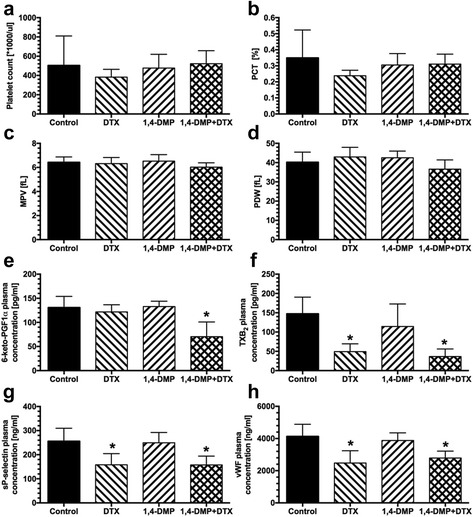
The influence of the combined treatment comprising the use of docetaxel (DTX) and 1,4-DMP on platelet morphology and activity in BALB/c Nude mice bearing PC-3M-luc2 tumors. **a** Platelet count; **b** platelet crit (PCT); **c** mean platelet volume (MPV); **d** platelet distribution width (PDW) determined on the last day of the experiment (day 46). Plasma concentration of: **e** 6-keto-prostaglandin F1α (6-keto-PGF1α) (*p < 0.05 vs. control and 1,4-DMP); **f** thromboxane B_2_ (TXB_2_) (*p < 0.05 vs. control and 1,4-DMP); **g** soluble P-selectin (*p < 0.05 vs. control and 1,4-DMP); and **h** von Willebrand Factor (vWF) (*p < 0.05 vs. control and 1,4-DMP), determined on the last day of the experiment (day 46). All data are presented as mean ± SD values

The analysis of the morphological parameters of blood platelets revealed that while docetaxel given alone slightly lowered the platelet count, 1,4-DMP did not influence the platelet number when given alone but restored the number in docetaxel-treated animals (Fig. 3a and b). In addition, we observed that in animals receiving 1,4-DMP together with docetaxel, the mean platelet volume (MPV) and PDW were lowered when compared to the untreated animals (6.02 ± 0.4 and 36.6 ± 4.8 vs. 6.42 ± 0.4 and 40.19 ± 5.2 fL in the control group of animals, Fig. 3c and d). In addition, when analyzing biochemical parameters of platelet activity, we observed that both docetaxel given alone as well as administered with 1,4-DMP significantly reduced plasma concentrations of TXB_2_ (49.18 ± 24.0 pg/ml, 36.55 ± 19.6 pg/ml, respectively, vs. 147.4 ± 43.1 pg/ml in the control, *p* < 0.05), soluble P-selectin (158.3 ± 46.3 ng/ml, 157.6 ± 36.7 ng/ml, respectively, vs. 256 ± 53.9 ng/ml in the control, *p* < 0.05), and vWF (2479 ± 764 ng/ml, 2785 ± 432 ng/ml, respectively, vs. 4134 ± 753 ng/ml in the control) (Fig. 3f–h). However, we also observed a significant drop in 6-keto-PGF1α plasma concentration in mice treated with both docetaxel and 1,4-DMP (70.36 vs. 130.7 pg/ml, *p* < 0.05) (Fig. 3e).

### Toxicity of the combined treatment of prostate cancer comprising simultaneous application of 1,4-DMP and docetaxel

**Fig. 4:**
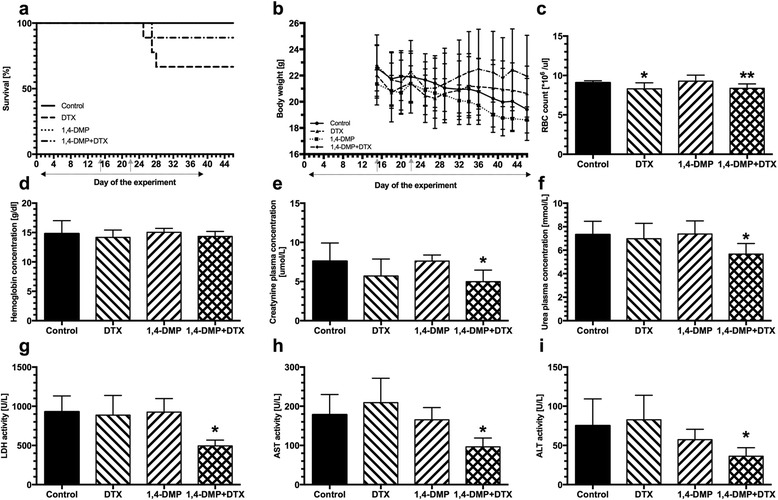
Toxicity of the combined treatment of prostate cancer comprising simultaneous application of 1,4-DMP and docetaxel (DTX). **a** Survival of BALB/c Nude mice bearing PC-3 M-luc2 tumors, treated with docetaxel (DTX) and 1,4-DMP either alone or in combination. **b** Body weight of BALB/c Nude mice bearing PC-3M-luc2 tumors, treated with docetaxel (DTX) and 1,4-DMP either alone or in combination. In graphs a and b, days of drug administration are indicated with *gray arrows* for docetaxel (DTX) and *dotted arrow* for 1,4-DMP. **c** Red blood cell (RBC) count (*p < 0.05 vs. 14-DMP; **p < 0.05 vs. control and 1,4-DMP); and **d** hemoglobin concentration in blood samples taken from BALB/c Nude mice bearing PC-3M-luc2 tumors on the last day of the experiment (day 46). Plasma concentrations of: **e** creatinine (*p < 0.05 vs. control and 1,4-DMP) and **f** urea (*p < 0.05 vs. control and 1,4-DMP) determined for plasma samples obtained from BALB/c Nude mice bearing PC-3M-luc2 tumors on the last day of the experiment (day 46). The activity of: **g** lactate dehydrogenase (LDH) (*p < 0.05 vs. control, DTX and 1,4-DMP); **h** aspartate aminotransferase (AST) (*p < 0.05 vs. control, DTX and 1,4-DMP); and **i** alanine aminotransferase (ALT) (*p < 0.05 vs. control and 1,4-DMP) determined in blood plasma samples obtained from BALB/c Nude mice bearing PC-3 M-luc2 tumors on the last day of the experiment (day 46). All data are presented as mean ± SD values

In the control and 1,4-DMP-treated groups of animals, there were no cases of deaths recorded. On the contrary, administration of docetaxel resulted in 3 incidences of treatment-related deaths (effect was not statistically significant). Surprisingly, docetaxel-induced toxicity was lowered when cytotoxic drug was given simultaneously with 1,4-DMP. In case of animals treated with the combined regimen, only 1 incidence of death was recorded (Fig. 4a). Regardless of the treatment applied, the body weight of all tumor-bearing BALB/c Nude mice was decreasing throughout the study with the most prominent body loss observed among control and 1,4-DMP-treated animals (approx. 10–12% body weight loss). Among the mice treated with docetaxel, a reduced body loss was observed (approx. 6% body weight loss), which was nearly abolished among animals treated with the combined treatment (approx. 3% body weight loss) (Fig. 4b).

The analysis of blood morphology of the treated animals revealed that similar to the platelet count described above, red blood cell count was lower in mice receiving docetaxel alone (8.30 ± 0.8 × 10^6^ vs. 9.1 ± 0.2 × 10^6^). However, in contrast to platelet count, red blood cell count was not restored when docetaxel was administered simultaneously with 1,4-DMP (8.4 ± 0.6 × 10^6^) and corresponded to non-significant change in hemoglobin content that was observed in mice given docetaxel either alone or in combination with 1,4-DMP (14.17 ± 1.3 g/dl and 14.34 ± 0.9 g/dl vs. 14.81 ± 2.2 g/dl in the control).

Blood biochemistry analysis revealed that in groups receiving docetaxel, levels of plasma concentrations for creatinine (5.7 ± 2.2 vs. 7.59 ± 2.3 μmol/l in the control) and urea (6.98 ± 1.3 vs. 7.33 ± 1.1 mmol/l in the control) were not significantly changed. However, when docetaxel was concurrently administered with 1,4-DMP, it resulted in a significantly lowered creatinine (4.99 ± 1.5 μmol/l, *p* < 0.05 vs. control and 1,4-DMP-treated group) and urea (5.67 ± 0.9 mmol/l, *p* < 0.05 vs. control and 1,4-DMP-treated group) concentrations (Fig. 4e–f).

On the contrary, as it was determined for the blood plasma samples taken from the treated mice bearing PC-3M-luc2 tumors, administration of docetaxel had no influence on the activity of lactate dehydrogenase (LDH) (887.1 ± 251.5 vs. 929.6 ± 202.5 U/l in the control, Fig. 4g) while resulted in a slightly increased activity of aspartate aminotransferase (AST) (209.1 ± 62.4 vs. 178.3 ± 51.7 U/l in the control) and alanine aminotransferase (ALT) (82.72 ± 31.8 vs. 75.23 ± 34.1 U/l in the control group) (Fig. 4h and i). However, when docetaxel was given simultaneously with 1,4-DMP to mice, significantly lower activity of all studied liver enzymes was observed (LDH: 494.2 ± 73.9 U/l; AST: 96.36 ± 22.6 U/l; for both enzymes *p* < 0.05 vs. control, docetaxel and 1,4-DMP-treated group; ALT: 36.16 ± 10.9 U/l, *p* < 0.05 vs. control and DTX-treated group) (Fig. 4g–i).

## Discussion

1-MNA is an endogenous metabolite of NA that was previously shown to possess significant anti-inflammatory and anti-thrombotic activity [17, 18]. 1-MNA is synthetized by nicotinamide N-methyltransferase (NMMT), an enzyme expressed primarily in liver cells where it participates in methylation of NA and other pyridine compounds [24]. Concurrently, the expression of NMMT was reported in multiple types of cancer in which it was associated with tumor-promoting activity [25–27] that could be further attributed to 1-MNA [28]. On the contrary, some of the published reports show the beneficial correlation between NMMT expression and cancer survival [29, 30]. Along the lines with such data in a recently published study, we have shown that exogenous 1-MNA does not enhance the growth of cancer cells neither in vitro nor in vivo but, in contrary, may possess antimetastatic activity, most likely resulting from its PGI_2_-releasing capacity. We have also shown that 1,4-DMP, a structural analog of 1-MNA, possesses similar antimetastatic activity; however, both compounds seemed to have different mechanisms of action that ultimately resulted in platelet-dependent metastasis inhibition. Importantly, pyridine compounds, but particularly 1,4-DMP, when given in a combination with cyclophosphamide contributed to its anticancer activity enhancing both antitumor and antimetastatic activity of cytostatic drug [21]. Referred studies were, however, carried out exclusively in the mouse model of breast cancer, and the report did not mention any activity of 1-MNA or 1,4-DMP in other types of malignant tumors or with different anticancer agents.

In the present work, we investigated the activity of both compounds in the model of TRAMP mice that spontaneously develop prostate tumors. Similar to our previously reported studies [21], both compounds, when administrated alone to mice developing prostate tumors, revealed no significant anticancer activity; however, to some extent these compounds delayed the disease onset (Fig. 1a–c). Such a delayed disease onset might be attributed to the prostacyclin-dependent activity of the compounds, as prostacyclin was shown to inhibit lung tumor development in PPARγ-dependent mechanism [31] that was also shown to be involved in tumor growth arrest in prostate tumors [32, 33]. Lack of the significant tumor preventing activity of both pyridine compounds while being somehow disappointing in terms of the possible application of 1-MNA, and its analog in the prevention of prostate cancer, is important for their implementation in anticancer treatments, in general, as once again we demonstrated that neither 1-MNA nor 1,4-DMP promoted the growth of solid tumors. On the contrary, we have not observed any antimetastatic activity of neither of the studied compounds. In contrast, among 1-MNA-treated animals, we even observed a slight increase of metastases frequency (Fig. 1c and d). Such a surprising result might be the consequence of prostacyclin-related inhibition of natural killer cells [34] that, in turn, was shown to stimulate prostate tumor metastasis [35]. Another explanation of the observed limited antimetastatic activity of both 1-MNA and 1,4-DMP might be associated with previously reported relationship between thrombin generation and the growth and metastasis of prostate tumors in TRAMP mice [36]. Possibly, thrombin as a potent coagulation and platelet activator that was proven to facilitate metastasis [37] counteracts a possible antiplatelet activity of pyridine compounds in this model. Importantly, in this study, we noted that during the prostate tumor development, TGF-β1 plasma concentration in TRAMP mice increased (Fig. 1p), which remains consistent with the previous reports indicating the usefulness of this molecule as a prognostic factor in prostate tumors [38].

When investigating the anticancer activity of therapeutic regimen including simultaneous use of docetaxel and 1,4-DMP (that seemed to be more potent in the model of prostate tumor as compared with 1-MNA) in the therapy of metastatic human prostate cancer PC-3M-luc2, we observed 60% enhancement of the antitumor activity of docetaxel given alone (Fig. 2c) and complete abolition of metastases formation (Table 3) in mice treated with docetaxel administrated with 1,4-DMP. These results confirm that 1,4-DMP may promote anticancer activity of various cytotoxic drugs. Beneficial therapy outcome was also reflected in the decreased plasma level of TGFβ-1, a molecule often acknowledged as a prognostic marker in prostate cancer (Fig. 2i).

TGF-β1 is commonly recognized as a molecule inducing epithelial-to-mesenchymal transition (EMT) in tumor-forming cells. EMT is a phenomenon in result of which non-invasive tumor cells of epithelial phenotype acquire mesenchymal properties and become able to migrate and invade distant tissues [39]. Therefore, in our study, lower plasma concentration of TGF-β1, and by implication lower metastatic capacity, was associated with higher expression ratio of E-cadherin to N-cadherin (Fig. 2f), cell adhesion molecules commonly accepted as important markers of EMT in cancer cells, including those of prostate origin [40]. Lower metastatic capacity of tumor-forming cells was additionally accompanied by the lower level of short E-cadherin fragments (40 kDa) observed in the tumor mass of mice lacking metastases. Such short intracellular protein fragments arise because of full-length E-cadherin cleavage resulting in the release into extracellular matrix and next to bloodstream of 80 kDa E-cadherin extracellular domain [41]. Indeed, 80 kDa fragments identified in metastatic sites or serum were previously discussed as potential prostate cancer progression markers [42, 43]. Accordingly, in our study, we observed that 40 kDa intracellular domain of E-cadherin was abundant in tumor mass isolated from mice bearing PC-3M-luc2 tumors diagnosed with metastases (Fig. 2g). Finally, the prominent efficacy of the combined treatment comprising the use of docetaxel and 1,4-DMP is additionally confirmed by the decreased expression of VEGFR-1 (Fig. 2h), another prognostic marker that has been previously linked to enhanced metastatic potential of prostate tumors [44].

We have previously shown that observed enhanced antitumor and antimetastatic activity of cytotoxic drugs when in a combination with 1,4-DMP might be a result of anti-platelet activity of the latter compound [21]. Platelets, in turn, contribute to metastases formation by several mechanisms as comprehensively reviewed in the literature [45, 46]. To confirm that increased anticancer efficacy of docetaxel observed when cytotoxic drug was given with 1,4-DMP was associated with diminished platelets activity, we have analyzed morphological and biochemical parameters reflecting platelet activation status. In this regard, in mice receiving the studied combined treatment, we observed lowered values of mean platelet volume and PDW (Fig. 3a–d) that may suggest decreased platelet activity [47, 48]. Additionally, in mice treated with docetaxel and 1,4-DMP, we also noted a marked reduction in plasma concentrations of TXB_2_, vWF and soluble P-selectin (Fig. 3e–h), constituting biochemical markers, further confirming diminished platelet activity.

Interestingly, increased antitumor activity of docetaxel when administrated simultaneously with 1,4-DMP was accompanied by its reduced toxicity manifested in the decreased incidence of treatment-related deaths and improved liver function (Fig. 4). Although we are currently investigating these phenomena, our initial results indicate that the observed protective activity of 1,4-DMP may involve acetylcholinesterase and consequently histamine-dependent pathways. It seems possible that in response to the treatment with 1,4-DMP, the level of histamine is increased that, in turn, may prevent liver injury [49]. This novel and unexpected feature of the 1,4-DMP treatment might not only be of a great value for possible improvement of side effects in patients undergoing chemotherapy, but may also allow to increase dosages in patients with drug-resistant tumors to induce desired response while maintaining acceptable treatment toxicity.

## Conclusions

The results of the presented study prove that neither 1-MNA nor 1,4-DMP when administrated alone do not influence the development and growth of the primary prostate tumors supporting our previous findings in the murine model of metastatic breast cancer. However, pyridine compounds, such as 1,4-DMP, may beneficially influence the antitumor and antimetastatic activity of docetaxel and additionally limit the side effects accompanying chemotherapy. Such findings allow us to believe that pyridine compound endowed with PGI_2_ releasing properties [18, 20] may become a promising agent for the adjuvant therapy of metastatic cancer.

## Additional file

Additional file 1: Figure S1. Changes in blood morphology and TGF-β1 plasma concentration in TRAMP mice over time. Analysis were carried out on blood samples taken on days 87, 122, 213 of the experiment and during the necropsy of the animals: **a** platelet count (**p* < 0.05 vs. control D213, ***p* < 0.05 vs. 1,4-DMP D122); **b** mean platelet volume (MPV); **c** platelet distribution width (PDW) (**p* < 0.05 vs. Control D87, D122, D213; ***p* < 0.05 vs. 1,4-DMP D87, D122); **d** red blood cell count (**p* < 0.05 vs. Control D87, D213, ** *p* < 0.05 vs. 1-MNA D87, ****p* < 0.05 vs. 1,4-DMP D87, D213); **e** lymphocyte count (**p* < 0.05 vs. Control D87; ***p* < 0.05 vs. 1,4-DMP D87, D213); **f** monocyte count; **g** granulocyte count and **h** TGF-β1 determined by ELISA (**p* < 0.05 vs. Control D122, D213’ ***p* < 0.05 vs. 1-MNA D87, D213; ****p* < 0.05 vs. 1,4-DMP D87, D213). All data are presented as mean ± SD. Data significantly different (*p* < 0.05) are marked with stars. (TIFF 888 kb)

## Abbreviations

%BW: % of body weight
1,4-DMP: 1,4- dimethylpyridine
1-MNA: 1-methylnicotinamide
6-keto-PGF1α: 6-keto-prostaglandin F1α
a.u.: Arbitrary units
ALT: Alanine aminotransferase
AST: Aspartate aminotransferase
DTX: Docetaxel
EDTA: Ethylenediaminetetraacetic acid
EMT: Epithelial to mesenchymal transition
HBSS: Hanks Balanced Salt Solution
LDH: Lactate dehydrogenase
MPV: Mean platelet volume
NA: Nicotinamide
NNMT: Nicotinamide N-methyltransferase
PCT: Platelet crit
PDW: Platelet distribution width
PGI_2_: Prostacyclin
PVDF: Polyvinylidene fluoride
RBC: Red blood cells
rpm: Round per minute
SD: Standard deviation
SDS: Sodium dodecyl sulfate
TGF-β1: Transforming growth factor β1
TGI: Tumor growth inhibition
TNF-α: Tumor necrosis factor α
TV: Tumor volume
TXB_2_: Thromboxane B_2_
VEGFR-1: Vascular endothelial growth factor receptor 1
vWF: von Willebrand Factor
