# Supplementary figures and images for: The effects of 1,4-dimethylpyridine in metastatic prostate cancer in mice
Source: BMC Cancer. 2017 Mar 7;17:177. doi: 10.1186/s12885-017-3161-4 (PMC5341170; doi:10.1186/s12885-017-3161-4)

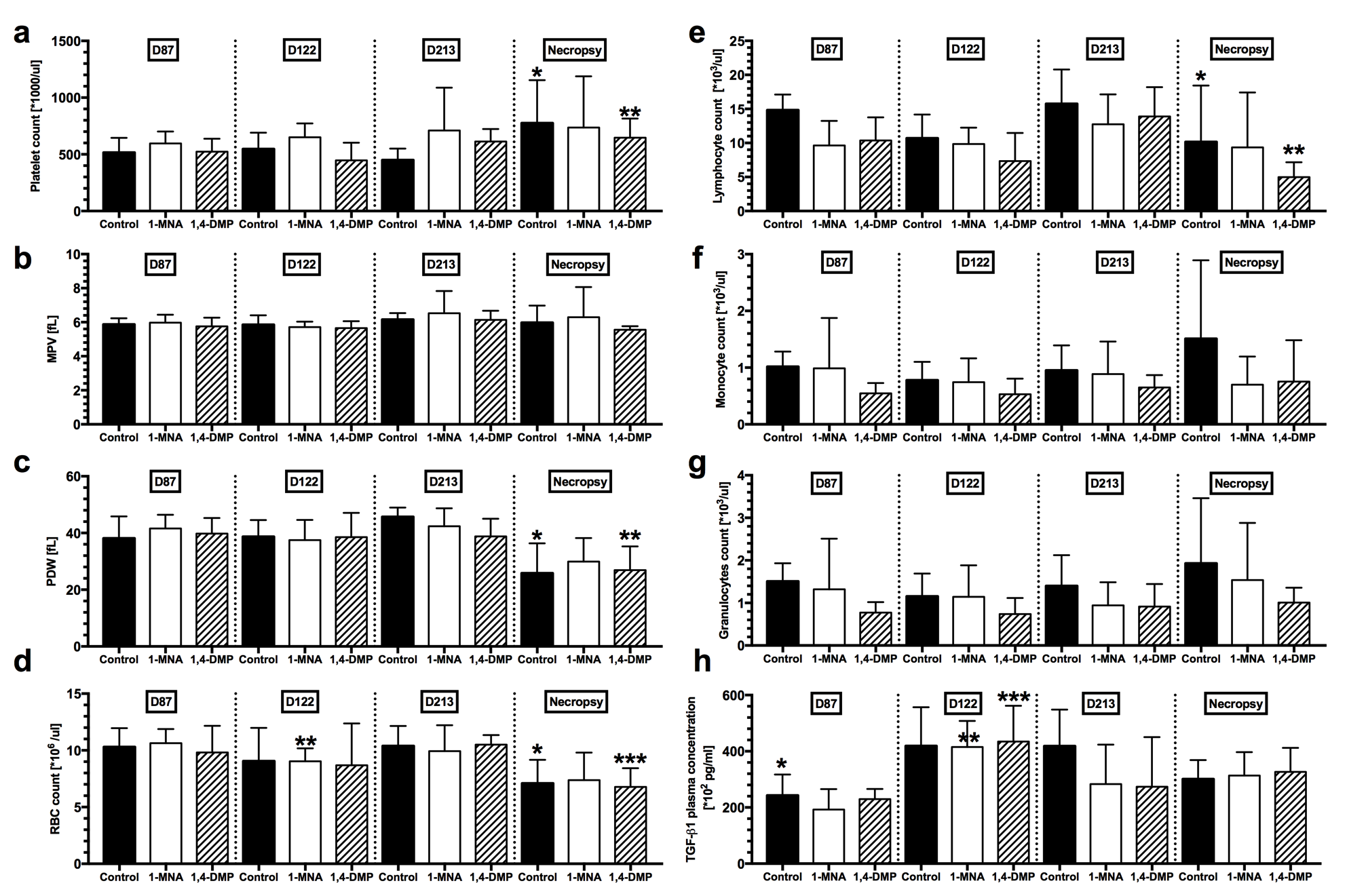

Supplement: Additional file 1: Figure S1. — Changes in blood morphology and TGF-β1 plasma concentration in TRAMP mice over time. Analysis were carried out on blood samples taken on days 87, 122, 213 of the experiment and during the necropsy of the animals: a platelet count (*p < 0.05 vs. control D213, **p < 0.05 vs. 1,4-DMP D122); b mean platelet volume (MPV); c platelet distribution width (PDW) (*p < 0.05 vs. Control D87, D122, D213; **p < 0.05 vs. 1,4-DMP D87, D122); d red blood cell count (*p < 0.05 vs. Control D87, D213, ** p < 0.05 vs. 1-MNA D87, ***p < 0.05 vs. 1,4-DMP D87, D213); e lymphocyte count (*p < 0.05 vs. Control D87; **p < 0.05 vs. 1,4-DMP D87, D213); f monocyte count; g granulocyte count and h TGF-β1 determined by ELISA (*p < 0.05 vs. Control D122, D213’ **p < 0.05 vs. 1-MNA D87, D213; ***p < 0.05 vs. 1,4-DMP D87, D213). All data are presented as mean ± SD. Data significantly different (p < 0.05) are marked with stars. (TIFF 888 kb) [file 12885_2017_3161_MOESM1_ESM.tiff]
